# Organismic Memristive Structures With Variable Functionality for Neuroelectronics

**DOI:** 10.3389/fnins.2022.913618

**Published:** 2022-06-14

**Authors:** Natalia V. Andreeva, Eugeny A. Ryndin, Dmitriy S. Mazing, Oleg Y. Vilkov, Victor V. Luchinin

**Affiliations:** ^1^Department of Micro- and Nanoelectronics, Faculty of Electronics, Saint Petersburg State Electrotechnical University “LETI”, Saint Petersburg, Russia; ^2^Department of Solid State Electronics, Saint Petersburg State University, Saint Petersburg, Russia

**Keywords:** analog non-volatile and volatile tuning of the resistance, emulation of synaptic plasticity and neural activity, nanolayered memristive compositions, multilevel memristor, atomic layer deposition

## Abstract

In this paper, we report an approach to design nanolayered memristive compositions based on TiO_2_/Al_2_O_3_ bilayer structures with analog non-volatile and volatile tuning of the resistance. The structure of the TiO_2_ layer drives the physical mechanism underlying the non-volatile resistance switching, which can be changed from electronic to ionic, enabling the synaptic behavior emulation. The presence of the anatase phase in the amorphous TiO_2_ layer induces the resistive switching mechanism due to electronic processes. In this case, the switching of the resistance within the range of seven orders of magnitude is experimentally observed. In the bilayer with amorphous titanium dioxide, the participation of ionic processes in the switching mechanism results in narrowing the tuning range down to 2–3 orders of magnitude and increasing the operating voltages. In this way, a combination of TiO_2_/Al_2_O_3_ bilayers with inert electrodes enables synaptic behavior emulation, while active electrodes induce the neuronal behavior caused by cation density variation in the active Al_2_O_3_ layer of the structure. We consider that the proposed approach could help to explore the memristive capabilities of nanolayered compositions in a more functional way, enabling implementation of artificial neural network algorithms at the material level and simplifying neuromorphic layouts, while maintaining all benefits of neuromorphic architectures.

## Introduction

Neuromorphic electronics is based on algorithms and rules of information processing similar to the biological neural networks operation. Such an imitation concerns mainly the architecture level (Intel Loihi, IBM True North) (Merolla et al., 2014; Davies et al., 2018), and for building the optimal topology, the basic blocks are used, reproducing the functionality of synapses and neurons of a biological neural network. To increase the efficiency of the artificial neural network (ANN) algorithms and reduce power consumption, crossbar arrays with memristive structures are commonly applied. They act as synapses under the hardware implementation and make it possible to sufficiently simplify the matrix-vector multiplication, which is a key ANN operation. Non-volatile, analog switching of the resistance in memristive devices simulates the weight changing of synapses when the signal transmission through the neural network occurs. Successful hardware implementation of the described neuromorphic systems for the purposes of artificial intelligence was demonstrated for thin-film metal oxide compositions based on TiN/TaO_*x*_/HfO_*x*_/TiN (up to 32 resistance states) (Yao et al., 2020) and Pt/Al_*x*_O_*y*_ /TiO_2_/Pt (up to 92 resistance states) (Stathopoulos et al., 2017) structures.

The operation of such memristive structures is determined by the characteristics of an active, several nanometers-thick layers (HfO_*x*_ or Al_*x*_O_*y*_), providing changes in the resistance level of the structure, and a layer of TaO_*x*_ or TiO_2–x_, which has a thickness up to several tens of nanometers and acts as an oxygen vacancies reservoir. In some cases, to govern the concentration of oxygen vacancies in the active layer, oxidation of one of the device electrodes can be used by applying the external voltage. In particular, in a memristive structure of Ta/HfO_2_/Pt (Xia et al., 2019), 64 intermediate resistive states are achieved due to electromigration of tantalum cations into the layer of hafnium oxide and oxygen anions into the tantalum electrode. Thus, oxygen vacancies appear in the active switching layer. In this case, tantalum acts as a dopant for the hafnium oxide layer, and when its concentration increases, a conductive channel between the electrodes is established. The formation of intermediate resistive states occurs at a variable ratio between the tantalum ions and the oxygen vacancies in the conducting channel, while the switching between the different states is achieved by varying the current compliance or the number of voltage pulses applied to the structure. Obviously, control over the concentration of ions in the active layer of thin-film memristive structures *via* redox processes or electromigration is essential for the successful implementation of electronic analogs of synapses with a gradient resistive switching.

At the same time, modern computing systems, regardless of their architecture, are built on digital transistor logic, which is based on purely electronic processes. Well-developed digital approaches and transistor logics are used in an effort to implement the functional capabilities of synapses and neurons in analogy with the bioelectrogenesis based on ionic processes and known algorithms for the operation of neural networks. In other words, the emulation of neural networks is performed with an electronic yardstick. Simultaneously, the demands for the neuromorphic (or memristor) elements are determined by the success of their integration with the transistor logic under the hardware implementation of spike neural network algorithms.

A closer look at the list of the requirements reveals their poor compatibility with the physical principles, underlying the operation of memristive structures with analog resistance tuning based on ionic mechanisms:

1. The claim for reduced variability of the operating parameters of memristive structures. Increasing the size of crossbar arrays unacceptably widens the spread of the operating parameters of memristive elements, hindering their use in neuromorphic and computing systems.
2. The requirement for linearity of switching characteristics of memristive structures. As is known, the hardware implementation of multilayered neural networks on the basis of memristor logic provides recognition accuracy of 92% with 256 resistance levels (8-bit memristor cell) and up to ≈86% with 64 levels (6-bit cell) (Shi et al., 2018), being far inferior to the software-implemented neural network algorithms that provide recognition accuracy at the level of 90–99%. Thus, from an algorithmic point of view, to introduce the required number of resistive states, the possibly widest range of resistance is demanded. This is accompanied, however, by enhanced non-linearity of the switching characteristics, which makes it difficult to use algorithms for processing the signals propagating through the neural network. Attempts to compensate for the non-linearity by circuitry solutions complicate sufficiently the hardware implementation of neural networks.
3. The requirement for the conservation (stability) of the resistive state in time. When the resistive state is set by the distribution/concentration of ions in the active layer of the memristive structure, a crucial point is a rather high activation barrier for oxygen vacancy migration [3.9–6 eV in amorphous alumina with the formation energy of about 8 eV (Liu et al., 2010; Yang et al., 2013)]. A variation of the potential caused by the presence of structural defects is assumed to significantly reduce the activation energy of vacancy migration. In this case, the intermediate resistive states will inevitably relax with time. Thus, decreasing power consumption of memristive structures based on thin-film oxide layers correlates inversely with the stability of the resistive state in time.
4. Power consumption requirement. The issue of energy consumption under the integration of memristive elements into very-large-scale integration (VLSI) circuit remains relevant even for memristive structures based on solid electrolytes (CBRAM or ECM), in which the resistive switching is associated with the electrochemically governed growth and dissolution of the local conductive region, consisting of ions from an active electrode, into the oxide/chalcogenide matrix. In particular, for ECM cells, if the size of the narrowest part of the conductive metal filament in the oxide matrix changes by one atom, the conductivity will be modified by *G*_0_=2*e*^2^/*h*≈80μ*S*. The estimates show that at operating write voltages of 1–3 V (typical for copper or silver active electrodes), such modification corresponds to an increase in the programming current level by *I*_*prog*_≈*G*_0_(1−3*V*) = 80−240μA (Shi et al., 2018). With programming pulse durations of 10–100 ns, the power consumption per write operation in an ECM cell is 1–100 pJ, satisfying poorly the power consumption requirements for the subsequent integration into VLSI circuit.

In line with the above findings, all attempts to advance the multilevel logic at the hardware implementation of neuromorphic circuits with memristor elements, based on purely ionic processes, led to the widespread perception that such materials are of purely academic interest with the absence of serious technological prospects. Here, we propose to take a fresh look at the design of nanolayered memristive structures. We show that the same composition can provide a functional analog of a synapse, both due to electronic and ionic processes, wherein the transition between them is governed by the physical parameters specified during the synthesis of the functional layers. In our opinion, the reliance on electronic processes should provide smooth integration of memristive structures into circuits of neuromorphic computing architectures at a given paradigm of circuit engineering approaches in modern electronics.

## Materials and Methods

**FIGURE 1:**
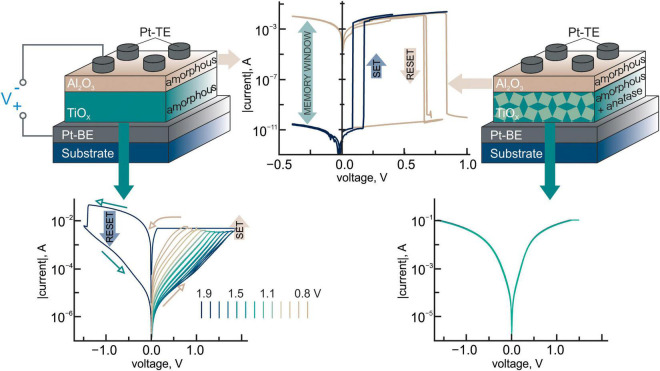
Schematic illustration of Pt/TiO_2_/Al_2_O_3_/Pt bilayer structures with amorphous titanium dioxide layer (on the left) and bilayer structures with amorphous titanium dioxide layer with the inclusion of the anatase phase crystallites (on the right). The experimental I-V characteristics of Pt/TiO_2_/Pt structures with gradual resistance tuning in the range of three orders of magnitude (on the left) and without any resistive effects (on the right). Experimental I-V characteristics of the resistive switching in Pt/Al_2_O_3_/Pt structures determining the memory window in the bilayers of seven orders of magnitude are shown in the middle.

As a memristive structure with an analog switching of the resistive state, a sequence of two Al_2_O_3_/TiO_2_ thin-film layers was chosen (Figure 1). To form the structures, Pt bottom electrode (100 nm) was deposited at 150°C on a p-Si/SiO_2_ substrate with an adhesive layer of Ti (25 nm). Atomic layer deposition (ALD) was used to synthesize the functional oxide layers (TFS 200 setup, Beneq). ALD of 30-nm-thick titanium dioxide was carried out at temperatures of 150–200°C using titanium tetraisopropoxide [Ti(OiPr)_4_] and water vapor (H_2_O) as precursors. Synthesis of a functional 5-nm-thick Al_2_O_3_ layer was carried out at 150°C using trimethylaluminum and water vapor (H_2_O). The structure of the titanium dioxide layer solely depends on ALD temperature *T*_*c*_. At *T*_*c*_ = 150°C, the titanium dioxide layer is completely amorphous, whereas at *T*_*c*_ = 200°C, it is amorphous with the inclusion of the anatase phase crystallites (Grzegorz et al., 2013; Piltaver et al., 2017; Andreeva N. et al., 2018). The initial resistance of the amorphous layer was 10^9^ Ω⋅cm, and that of the layer, containing the anatase phase, was 10^4^ Ω⋅cm. The initial resistance of the two-layer Al_2_O_3_/TiO_2_ structures was determined by the resistance of the amorphous aluminum oxide layer and was set to 10^11^–10^12^ Ω⋅cm.

The thickness of the deposited layers was controlled with scanning electron microscopy over the cross-section of the structures obtained with a focused ion beam (Quanta FEI, Helios NanoLab). The surface morphology of platinum and titanium oxide films was studied with atomic force microscopy (Dimension 3100, Veeco). The study of local resistive properties was carried out in the mode of tunneling atomic force microscopy using probes with a conductive platinum coating.

Top platinum and copper electrodes with a thickness of 100 nm and a diameter of 350 μm were deposited through a mask by ion-plasma sputtering at 150°C. Mechanisms responsible for the appearance of resistive switching and the switching effect itself were studied based on I–V characteristics measured using the Keithley 4200-SCS semiconductor characterization system. During the measurements, the bottom platinum electrode was grounded.

Aluminum oxide was chosen as the active layer responsible for the analog switching of the resistance for the following reasons. First, it is an oxide of a non-transition metal, and the effect of redox reactions on the resistive switching processes may be excluded. Second, it is a high-resistance material [with a band gap of 6.2–6.5 eV for amorphous alumina (Liu et al., 2010)] with low charge carrier mobility and long dielectric relaxation time.

At a low concentration of defects (point defects, mainly in the oxygen sublattice), the conductivity of aluminum oxide is electronic in nature (Robert and Doremus, 2006). At that point, the hopping transport of electrons through the trap levels, arising from structural defects, and space-charge-limited currents (SCLC) act as the main mechanisms of charge transport (Hickmott, 1962; Kunitsyn et al., 2018). Taking into account the peculiarities of the ALD technology, it is possible to synthesize layers, in which both chemically bound and adsorbed (OH-) groups will be present in the bulk of the deposited Al_2_O_3_ layers. At a certain concentration of (OH-) groups, exceeding 3×10^7^ (OH-) groups per aluminum atom, a transition in the character of conductivity from electronic to ionic is observed. The ionic conductivity is related to the transport of H_3_O^+^ ions *via* the formation of bonds with oxygen anions located near the AlO defects (Robert and Doremus, 2006). ALD synthesis makes it possible to vary the concentration of OH- groups in Al_2_O_3_ layers by changing the exposure time to water. Thus, the use of ALD-grown aluminum oxide as functional layers in memristive structures allows us to vary the contribution of electronic or ionic processes in the mechanisms of resistive switching.

The use of titanium dioxide, which is a transition metal oxide, as the second functional layer in the structure of memristive composition gives us an opportunity to “optionally,” i.e., depending on the synthesis conditions, engage the mechanism of resistive switching related to the redox reactions. For example, a layer of amorphous titanium dioxide exhibited a reversible analog switching of the resistance in the range of three orders of magnitude due to the redox reactions (Andreeva et al., 2021), and it was used in Al_2_O_3_/TiO_2_ compositions with a predominance of the ionic resistive switching mechanism. No resistive effects were observed in the titanium dioxide layer with the anatase phase, and its use made it possible to turn to the electronic nature of resistive switching in two-layer memristive compositions (Alekseeva et al., 2016; Andreeva N. et al., 2018).

In the ionic switching mechanism, the replacement of platinum with copper as electrode material was aimed to govern the resistance of the aluminum oxide layer by varying the ratio of copper cations and oxygen vacancies.

## Results

**FIGURE 2:**
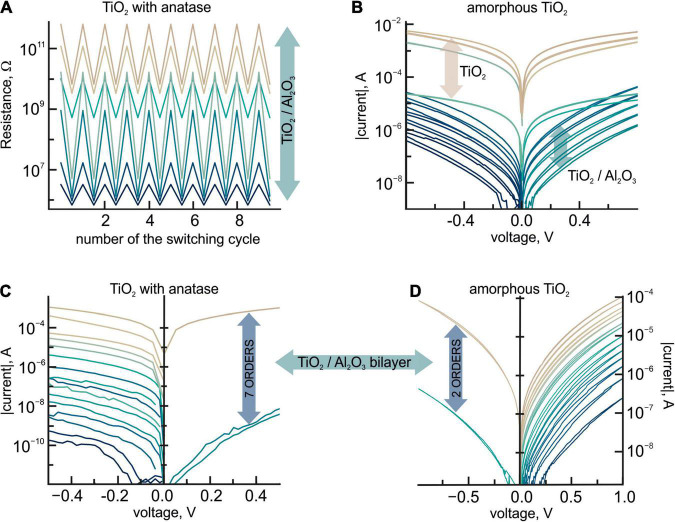
**(A)** R_*OFF*_/R_*ON*_ ratio for seven different resistance states of the Pt/TiO_2_/Al_2_O_3_/Pt bilayer structures with amorphous titanium dioxide layer with the inclusion of the anatase phase crystallites in the dependence on the switching cycle. The color on the figure corresponds to the resistance state of the bilayer structure, relative to which the coexistence of the bipolar resistance switching with the R_*OFF*_/R_*ON*_ ratio could be observed. The R_*OFF*_/R_*ON*_ ratio was determined for a bipolar resistive switching relative to the given resistance state for the Pt/TiO_2_/Al_2_O_3_/Pt structure [switching resistance from a high resistive state (HRS) to a low resistive state (LRS) happens when a negative voltage is applied to the Pt-TE], whereas the reset process (switching the resistance from an LRS to an HRS) occurs at a positive voltage (i.e., clockwise switching). The OFF-resistance/ON-resistance ratio for a certain resistance state is shown with a certain color. The transition between different resistance states was tuned in DC operation mode by current–voltage sweep in the range of –2.0 to –4.0 V. The resistance of the bilayer structure was measured by using a low (0.1 V) dc voltage. **(B)** The analog switching of the resistance state of the Pt/TiO_2_/Al_2_O_3_/Pt bilayer structures with amorphous titanium dioxide layer in the range of two orders of magnitude. An irreversible transformation of the aluminum oxide layer in the bilayer structures leads to a bipolar resistance switching typical for titanium dioxide shown above the range of the analog switching. **(C)** Experimental I-V characteristics of analog tuning of the resistance of Pt/TiO2/Al2O3/Pt bilayer structures with tiatanium dioxide layer with the inclusion of the anatase phase crystallites. **(D)** Experimental I-V characteristics of analog tuning of the resistance of Pt/TiO2/Al2O3/Pt bilayer structures with amorphous tiatanium dioxide layer.

Two-layer structures with a functional layer of titanium dioxide, containing an anatase phase, demonstrate multilevel resistive switching in the range of seven orders of magnitude when voltage pulses of negative polarity (from –2 to –4 V) are applied to the top electrode (Figures 2A,C). Using tunneling atomic force microscopy, we found the crystallites of the anatase phase to have resistive properties other than those of the amorphous phase of titanium dioxide (see Supplementary Material). It is noteworthy that the anatase phase is formed only during the synthesis of titanium dioxide on platinum. Changing the sequence of the layers in Al_2_O_3_/TiO_2_ compositions yields an amorphous structure of the titanium dioxide layer under the same technological conditions. Since the multilevel analog switching of the resistance in memristive compositions is absent at the reverse sequence of oxide layers (Alekseeva et al., 2016), the anatase phase plays a key role in the effects concerning the multilevel resistance tuning in Al_2_O_3_/TiO_2_ structures (Andreeva N. V et al., 2018). We observed the analog switching of the resistive state in Al_2_O_3_/TiO_2_ memristive compositions in a wide temperature range of 50–295 K (Andreeva N. et al., 2018) that indirectly confirmed the dominant role of electronic processes there. The non-linear dependence of the resistance level on the voltage applied is a feature of such compositions.

Rearrangement of the titanium dioxide layer in Al_2_O_3_/TiO_2_ structures, accompanied by changes in its resistive properties, modifies the way the analog resistive switching proceeds (Figures 2B,D). In particular, in two-layer structures with an amorphous, high-resistance layer of titanium dioxide, the polarity of the voltage, at which the multilevel analog switching occurs, is changed, and the operating range is shifted toward higher voltages (6–8 V). The tuning range of the resistance narrows down to 2–3 orders of magnitude, and the dependence of the resistance level on the voltage applied becomes almost linear. If the voltage exceeds some threshold value, an irreversible transformation of the aluminum oxide layer occurs, and the structure exhibits a bipolar switching between two non-volatile resistance states, which is typical for titanium dioxide. Memristive compositions with direct (Pt-BE/TiO_2_/Al_2_O_3_/Pt-TE) and reverse (Pt-BE/Al_2_O_3_/TiO_2_/Pt-TE) orders of oxide layers are symmetrical.

If copper is a material of the top electrode in memristive compositions, a region of negative differential resistance exists in the I-V characteristics. The tuning range of the resistance and operating voltages do not differ from those for the structures with platinum electrodes. However, a pronounced relaxation of the intermediate resistive states to the initial high-resistant level of the two-layer structures is observed.

## Discussion

At the electronic nature of conductivity in the ALD-deposited Al_2_O_3_ layer of memristive Pt-BE/TiO_2_/Al_2_O_3_/Pt-TE structures with an anatase phase in the titanium dioxide film, the main transport mechanisms are the hopping transport of electrons *via* trap levels and currents limited by space charge (SCLC) (Alekseeva et al., 2016; Andreeva et al., 2021). According to the results of *ab initio* modeling (Perevalov, 2015), the trap levels in aluminum oxide are related mostly to oxygen vacancies, and they are the donor centers. When an electron is trapped, initially neutral vacancies become negatively charged. Bearing in mind the clockwise switching of the resistive state in the structures, the top platinum electrode acts as an injector. For the SCLC mechanism, sufficiently high levels of carrier injection into the bulk of alumina is demanded. Such a mechanism manifests itself in the experiment as the power-law dependence of the current on the voltage (in the SCLC section), and the exponent strongly depends on the concentration of the trap centers, their energy, and spatial distribution.

**FIGURE 3:**
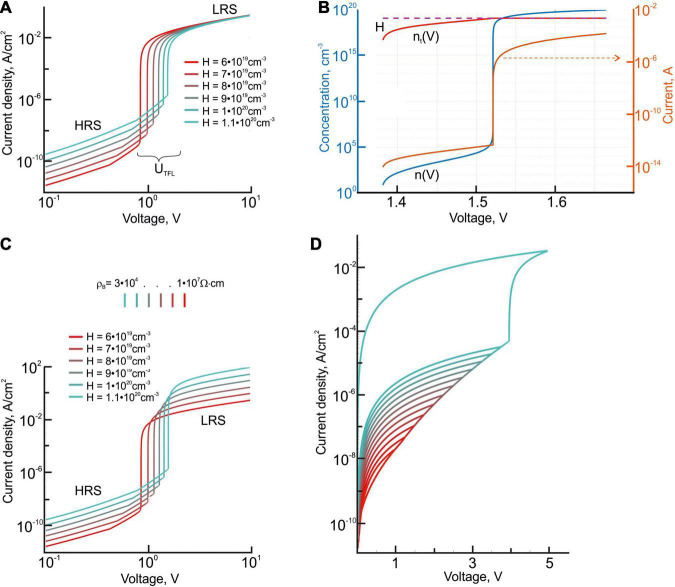
**(A)** I-V characteristics of the TiO_2_/Al_2_O_3_ bilayer structures with different concentrations of the trap centers H in the aluminum oxide layer. The trapping parameters were taken in accordance with (Perevalov, 2015): for *H* = 6⋅10^19^*cm*^3^, hopping transport activation energy *E*_*a*0_ = 1.9*eV* and *E*_*C*_−*E*_*t*_ = 1.7*eV*; for *H* = 7⋅10^19^*cm*^3^ - *E*_*a*0_ = 1.86*eV* and *E*_*C*_−*E*_*t*_ = 1.74*eV*; *H* = 8⋅10^19^*cm*^3^, *E*_*a*0_ = 1.82*eV*, *E*_*C*_−*E*_*t*_ = 1.78*eV*; *H* = 9⋅10^19^*cm*^3^,*E*_*a*0_ = 1.78*eV*,*E*_*C*_−*E*_*t*_ = 1.82*eV*; *H* = 1⋅10^20^*cm*^3^, *E*_*a*0_ = 1.74*eV*, *E*_*C*_−*E*_*t*_ = 1.86*eV*; *H* = 1.1⋅10^20^*cm*^3^, *E*_*a*0_ = 1.7*eV*,*E*_*C*_−*E*_*t*_ = 1.9*eV*. **(B)** The dependence of the non-equilibrium concentrations of electrons captured by traps (n_*t*_), non-equilibrium electron densities in the conduction band (n), and the current through the TiO_2_/Al_2_O_3_ bilayer structure on the applied voltage. **(C)** I-V characteristics of the TiO_2_/Al_2_O_3_ bilayer structures with different concentrations of the trap centers in the aluminum oxide layer. The resistance of the titanium oxide film, connected in series with the resistance of the active aluminum oxide film, varies in the range of 10^7^–3 × 10^4^ Ω⋅cm. **(D)** I-V characteristics of the TiO_2_/Al_2_O_3_ bilayer structures with the concentration of the trap centers in the aluminum oxide layer being equal to 8 ⋅ 10^19^ cm^– 3^. The gradual resistance tuning is associated with increasing the amplitude of the voltage pulses applied to the structure in the range of 1–5 V.

Having the voltage of the transition from ohmic conduction to SCLC, we can estimate the concentration of the trap centers in the aluminum oxide layers (for details, see Petrov et al., 2018), which is no less than 10^19^ cm^–3^. According to our model of electron transport in the active aluminum oxide layer, the switching of the resistance within the range of seven orders of magnitude (Andreeva N. et al., 2018), experimentally observed in single-layer Pt-BE/ALD-Al_2_O_3_/Pt-TE structures, is determined by the processes of trap filling in aluminum oxide and cannot be explained only by changing the concentration of trapping centers. A detailed description of the model is provided in the Supplementary Material. In particular, the resistance at the low-resistance state is not influenced by varying the concentration of the trapping centers in the range of 10^16^–10^20^ cm^–3^, but this variation leads to the shift of trap-filled limit voltage (U_*TFL*_) within one order of magnitude (Figure 3A). U_*TFL*_ increases with the increasing concentration of the trap centers. Notably, if the resistance switching occurs in single-layer Pt-BE/ALD-Al_2_O_3_/Pt-TE structures, the conductivity changes abruptly. According to the simulation, in Pt-BE/ALD-Al_2_O_3_/Pt-TE structures, a region with a sharp increase of the current is observed in the I-V curves when approaching the TFL mode (99% of the traps are filled). In this case, further injection of electrons strongly changes the concentration of free charge carriers *n* in the bulk of the film (Figure 3B), which is related to the concentration of electrons captured on the traps *n_t_* by the following equation:


(1) $$n=\frac{N\,n_{t}}{g\,\left(H-n_{t}\right)},$$

(2) $$n_{t}\,\left(E_{t}\right)=\frac{H}{1+\frac{1}{g\,\left(E_{t}\right)}\,e\,x\,p\,\left(\frac{E_{t}-E_{F}}{k_{B}\,T}\right)};$$

$$\,N\,\left(E_{t}\right)=N_{C}\,e\,x\,p\,\left(\frac{E_{t}-E_{C}}{k_{B}\,T}\right),$$

where, *N_C_* is the effective density of states in the conduction band of the active film, *E_t_* is the trap level, *E_C_* is the level of the bottom of the conduction band, *k_B_* is the Boltzmann constant, *T* is the temperature, *E_F_* is the Fermi level, *g*(*E*_*t*_) is the spin degeneracy factor, and *H* is the concentration of traps.

Upon reaching the TFL mode, a transition to the trapless SCLC takes place, and quadratic I-V characteristics are observed. In fact, the range of the current variation, corresponding to the abrupt increase of the current when approaching the TFL mode, is determined, according to Eqs 1, 2, by the band gap and the energy level of traps relative to the Fermi level. For the same mechanism of electron transport, the range of the resistance switching narrows down to 2–3 orders of magnitude for the materials with a smaller band gap (Petrov et al., 2018).

In two-layer Pt-BE/TiO_2_/Al_2_O_3_/Pt-TE structures with an anatase phase in the titanium dioxide layer, the resistance of the much thicker (6 times) titanium oxide film is connected in series with the resistance of the active aluminum oxide film. This gives a gentler slope of the I-V characteristics in the region of the abrupt increase of the current. This, in turn, makes it possible to implement the analog gradient tuning mode in two-layer structures, blurring the switching threshold to the range of about 0.5–1 V. Thus, gradually increasing the amplitude of the voltage pulses, applied to the two-layer structure, provides a gradient change of conductivity, which, in this case, is not always associated with the varying concentration of oxygen vacancies in the aluminum oxide film (Figures 3C,D).

In two-layer Pt-BE/TiO_2_/Al_2_O_3_/Pt-TE structures with amorphous titanium dioxide, the operating voltages of the gradient tuning of the resistive state increase. In particular, the resistive state changes at the applied voltage starting from 6 V. This value indicates indirectly the participation of ionic processes in the switching mechanism, since it correlates well with the activation energies of oxygen vacancy migration in Al_2_O_3_ [for a neutral vacancy, the activation energy varies in the range of 3.9–6.01 eV (Yang et al., 2013)] and with the formation energy of oxygen vacancy in titanium dioxide [4.85–4.2 eV for a neutral vacancy (Zavodinsky and Chibisov, 2009)]. As a rule, the formation of oxygen vacancies in amorphous oxide layers of memristive structures is associated with the redox reactions, involving oxygen-containing groups such as OH groups and molecular water (Robert and Doremus, 2006; Lübben et al., 2018; Valov and Tsuruoka, 2018).

Regarding the amorphous layer of aluminum oxide, it was found that the most probable mechanisms of diffusion and drift of molecular water and OH groups are the transfer of the hydroxonium ion H_3_O^+^ *via* its association with a negatively charged oxygen anion located near the AlO defect. In this case, the activation energy of diffusion of molecular water in aluminum oxide is 316 kJ/mol (Robert and Doremus, 2006). Therefore, if a positive voltage polarity is applied to the top platinum electrode of the two-layer structure, a drift of positively charged hydroxonium cations into the amorphous titanium dioxide layer will be observed.

In addition, negatively charged ions, such as *OH*^−^ and $O_{2}^{-2},$ may be present in the functional layer of titanium dioxide due to both the features of ALD synthesis and the possibility of the formation of oxygen vacancies. In this case, electrochemical reactions will take place at the interface of the two oxide layers with hydroxonium cations and negatively charged oxygen-containing groups being involved. The following reaction can be considered as one of the possibilities:


(3) $$2\,O\,H^{-}+2\,H_{3}\,O^{+}\to 4\,H_{2}\,O$$

The observed change in the concentration of OH groups in the layer of amorphous titanium dioxide is accompanied by the electrochemical reduction of Ti^4+^ to Ti^3+^ and transition to a lower resistive state.

Thus, we assume that when positive polarity is applied to the upper electrode of two-layer structures, two processes are simultaneously observed: the migration of hydroxonium ions from ALD-grown Al_2_O_3_ to the interface with the titanium dioxide layer, and modification of the stoichiometry in the titanium dioxide layer due to the formation of oxygen vacancies. Since the initial resistivity differs for aluminum oxide and titanium oxide layers, the resulting I-V curves at the initial stages of the resistive switching will be the sum of (i) the non-linear I-V characteristics of Al_2_O_3_ determined by the mechanisms of electron transport mentioned earlier, and (ii) the linear I-V characteristics of the titanium dioxide layer playing the role of ballast resistance. Decreasing the ballast resistance, caused by the increasing concentration of oxygen vacancies in the titanium dioxide layer, will lead to a steeper slope in the non-linear part of the I-V curve of the two-layer structures, which is in good agreement with our experimental results and model calculations.

It should be stressed that with positive polarity of the voltage applied to the top electrode of the structure, the injection of electrons into the aluminum oxide film occurs from the side of the titanium dioxide layer, and its level is clearly insufficient to enter the TFL mode. At the same time, taking the simulation results into account, we may assume that increasing the concentration of oxygen vacancies in the aluminum oxide layer will diminish the level of the high-resistance state of bilayer structures, which is determined exclusively by the resistance of the aluminum oxide film in the high-resistance state. Thus, starting from a certain voltage, in addition to the changing slope of the non-linear region in the I-V characteristics, an analog switching of the resistive state is experimentally observed for the bilayer structure. The switching is based on the varying concentrations of oxygen vacancies in the aluminum oxide layer but not on the filling of the trap levels, since the electron charge injected into the aluminum oxide layer will no longer be stored at the traps at applied voltages (Simmons and Verderber, 1967). The simulations demonstrate that a change in the concentration of the traps in aluminum oxide by three orders of magnitude is accompanied by a change in the level of the high-resistance state by two orders of magnitude. This explains the sharp narrowing of the switching range of the resistive state of bilayer structures with an amorphous layer of titanium dioxide. Thus, the predominance of ionic processes in the gradient switching of the resistive state in Pt-BE/TiO_2_/Al_2_O_3_/Pt-TE structures limits the range of the tuning by the value inherent to the high-resistance state of the titanium dioxide layer. As a rule, the transition to this state indicates an irreversible modification of the aluminum oxide layer. At the same time, the gradient switching in such structures remains possible, and it is similar to that observed in single-layer Pt-BE/TiO_2_/Pt-TE structures with the amorphous titanium dioxide layer (Andreeva et al., 2021), since it is related solely to the properties of this layer.

**FIGURE 4:**
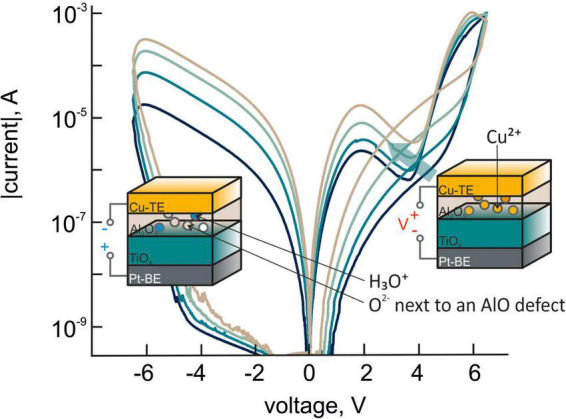
Experimental I-V characteristics of the TiO_2_/Al_2_O_3_ bilayer structures with copper electrodes exhibiting an N-shaped region.

There is a clear analogy with the processes of bioelectrogenesis. A layer of aluminum oxide is associated with a neuronal membrane, and its “permeability” (resistive state) is set by the concentration of oxygen vacancies, determined by the ratio between positively charged hydroxonium cations and negatively charged oxygen-containing groups at the interface with the second functional layer. Moving further along this line, we can assume that the addition of ions with a lower activation energy of the migration in the oxide layers of the structure (compared to that of the oxygen vacancies) should not only change the resistive state, but also simulate the excitation dynamics inherent to neuronal membranes. Indeed, the use of copper and platinum as electrode materials in combination with the oxide layers, containing OH groups, leads to the generation of EMF of up to 600 mV (Tappertzhofen et al., 2013). If we consider the EMF as a resting potential, the role of the action potential will be played by the impulse of the applied voltage, which changes the resistive state of the structure. Previously, we demonstrated (Andreeva et al., 2021) that the use of the copper electrodes, contacting the aluminum oxide layer in Pt-BE/TiO_2_/Al_2_O_3_/Cu-TE structures, makes it possible to create the structures with N-shaped I-V characteristics (Figure 4) that mimic the characteristics of excitable membranes. For these types of bilayer structures, contributions from the competition of oxygen vacancies and Cu ions into the formation of a conductive filamentary area in the Al_2_O_3_ layer are expected to take place. In this case, copper is considered as a volatile (mobile) dopant. The oxidation of the Cu electrode at the Al_2_O_3_/Cu–TE interface provides a higher migration rate of Cu ions due to the lower Cu–O bond energy compared to that of the Cu–Cu metallic bond (1.5 vs. 2.0 eV) (Ginnaram et al., 2020).

The appearance of the characteristic part of I-V curves with negative differential resistance is experimentally observed on the branch of I-V curves for a low-resistance state corresponding to the polarity of the reset process (Figure 4) at voltages comparable to the Cu–O bond energy. Thus, we assume that the N-shaped part of the I-V curves is related to the Cu ion migration in aluminum oxide and could be caused by the additional energy that is needed to break the bond between copper and AlO defects. Only after this activation energy is overcome that Cu ions can further respond to the voltage signal and move/switch faster (Lübben et al., 2020). In light of this assumption, the region of the I-V curve with a negative slope reflects the situation when the oxygen vacancy conductive filament dissolves faster than the Cu ion conductive filament forms. By analogy with neuronal membranes, where the appearance of the N-shaped region is caused by the different thresholds for voltage sensors of voltage-dependent channels (Cervera et al., 2016), the behavior of the I-V characteristics for Pt-BE/TiO_2_/Al_2_O_3_/Cu-TE structures is explained by the different activation energies of migration for the ions involved in the formation of the resistive state.

Thus, we may conclude that in memristive Pt-BE/TiO_2_/Al_2_O_3_/Cu-TE structures with the ionic mechanism of switching of the resistive state, two mandatory conditions for bioelectrogenesis are met: (1) existence of EMF, which is equivalent to the concentration gradients of electrolytes at the cell membrane, and (2) different activation energies of migration for ions of different types, which take part in the formation of the resistive state (or, by analogy, unequal permeability of an excitable membrane for ions contained in the living tissues).

## Conclusion

The results of modeling and experimental studies of resistive effects in memristive structures, composed of thin layers of titanium and aluminum oxides, indicate that the structure of oxide layers is responsible for the physical mechanism of analog resistive switching. Interestingly, despite the active layer, which governs the change of the state and its range, being the alumina thin film, the switching pattern is mainly determined by the properties of the titanium oxide layer. For example, the presence of conducting anatase phase in amorphous titanium dioxide ensures the electronic nature of the switching in the range of seven orders of magnitude, while the use of high-resistance amorphous titanium dioxide leads to the predominance of ionic processes in the observed resistive effects. In addition, applying the active electrode materials in these structures opens a way to use the same memristive composition both for emulating synaptic connections and as neurons themselves in neuromorphic architectures. The latter is ensured by the peculiarities in the drift of active electrode ions and oxygen-containing groups involved in the formation of oxygen vacancies in the functional layers of memristive compositions, setting together the type of I-V curve characteristic of excitable membranes.

Thus, the use of multilayered oxide memristive compositions makes it possible to move from the circuit implementation of the execution algorithms at the hardware organization of artificial neural networks toward a more complete utilization of the functionality of behavioral materials. In the future, such an approach will allow us to develop more flexible neuroelectronic analog architectures by applying the multifunctional element base. At the same time, the possibility of transition to the electronic mechanism of resistive switching in the memristive systems under consideration provides the firm background for the implementation of digital approaches to the construction of neuromorphic architectures.

## Data Availability Statement

The raw data supporting the conclusions of this article will be made available by the authors, without undue reservation.

## Author Contributions

NA and ER: conceptualization and visualization. ER: modeling. DM: technology and methodology. NA, OV, and VL: validation. NA and DM: investigation. NA and OV: writing—original draft preparation. DM and OV: writing—review and editing. VL: supervision. All authors have read and agreed to the published version of the manuscript.

## Conflict of Interest

The authors declare that the research was conducted in the absence of any commercial or financial relationships that could be construed as a potential conflict of interest.

## Publisher’s Note

All claims expressed in this article are solely those of the authors and do not necessarily represent those of their affiliated organizations, or those of the publisher, the editors and the reviewers. Any product that may be evaluated in this article, or claim that may be made by its manufacturer, is not guaranteed or endorsed by the publisher.
