# Supplementary material for: Organismic Memristive Structures With Variable Functionality for Neuroelectronics
Source: Front Neurosci. 2022 Jun 14;16:913618. doi: 10.3389/fnins.2022.913618 (PMC9238295; doi:10.3389/fnins.2022.913618)
Supplement: Supplementary file 1 [file Data_Sheet_1.docx]

Supplementary Material

**Organismic memristive structures with variable functionality for neuroelectronics**

**The theoretical analysis of stationary processes of charge carrier transport in Pt-BE / TiO_2_ (30 nm) / Al_2_O_3_ (5 nm) / Pt-TE structures**

The theoretical analysis of stationary processes of charge carrier transport in a bilayer memristive structure was performed using the following drift physical model:

| $J_{n}=q\mu_{n}n\mathcal{E}_{z}+J_{nt};$ | (1) |
| --- | --- |
| $\frac{\partial J_{n}}{\partial z}=0;$ | (2) |
| $\frac{\partial\mathcal{E}_{z}}{\partial z}=-\frac{q}{\varepsilon\varepsilon_{0}}\left( n-n_{0}+n_{t}-n_{t0} \right),$ | (3) |

where $n_{0}, n$ are equilibrium and non-equilibrium electron densities in the conduction band; $n_{t0}, n_{t}$ are equilibrium and non-equilibrium concentrations of electrons captured by traps; $J_{n}$ is a current density; $J_{nt}$ is a hopping current density component; $\mathcal{E}_{z}$ is an electric field in the active film (Al_2_O_3_); $\mu_{n}$ is an electron mobility in Al_2_O_3_; $\varepsilon_{T}$ is a relative permittivity of the active film; $\varepsilon_{0}$ is the vacuum permittivity; $q$ is the elementary charge; $z$ is the coordinate.

As a result of expressing the hopping current density $J_{nt}$ through the probability of filling the traps, described by the Fermi-Dirac statistics, taking into account the Frenkel-Pool effect, the original model (1) - (3) was reduced to the form:

| $J_{n}=q\mu_{n}n\mathcal{E}_{z}+sgn\left( \mathcal{E}_{z} \right)K_{n}\frac{n\cdot max\left\{ n,N\left( E_{t} \right)/g\left( E_{t} \right) \right\}}{\left( n+N\left( E_{t} \right)/g\left( E_{t} \right) \right)^{2}}sh\left( \sqrt{\frac{q^{3}\left\vert\mathcal{E}_{z} \right\vert}{\pi\varepsilon_{T}\varepsilon_{0}\left( k_{B}T \right)^{2}}} \right);$ | | (4) | |
| --- | --- | --- | --- |
| $K_{n}=2q\nu H^{2/3}exp\left( -\frac{E_{a0}}{k_{B}T}+\frac{k_{B}T}{\varepsilon_{p}} \right); N(E_{t})=N_{C}exp\left( \frac{E_{t}-E_{C}}{k_{B}T} \right);$ | | (5) | |
| $\frac{\partial\mathcal{E}_{z}}{\partial z}=-\frac{q}{\varepsilon\varepsilon_{0}}\left( n-n_{0}\left( E_{t} \right)+\frac{Hn}{n+{N\left( E_{t} \right)}/{g\left( E_{t} \right)}}-n_{t0}\left( E_{t} \right) \right);$ | (6) | |  |
| $V=J_{n}\rho_{B}d_{B}+\frac{k_{B}T}{q}ln\left( \frac{n_{C}}{n_{A}} \right)+\int_{0}^{d_{T}} \mathcal{E}_{z}(z)dz,$ | | (7) | |

where $H$ is a trap concentration; $N_{C}$ is an effective density of states in the conduction band of the active film; $E_{t}$ is a trap energy level; $E_{C}$ is a conduction band bottom level; $E_{a0}$ is a hopping transport activation energy; $\nu$ is a hopping attempt frequency; $\varepsilon_{p}$ is a parameter in energy units; $k_{B}$ is the Boltzmann's constant; $T$ is a temperature; $g\left( E_{t} \right)$ is a spin degeneracy factor; $d_{B}$ is a TiO_2_ film thickness; $d_{T}$ is a Al_2_O_3_ film thickness; $\rho_{B}$ is a TiO_2_ film resistivity; $n_{C}$, $n_{A}$ are electron densities at the boundaries of the Al_2_O_3_ film from the side of the cathode and anode.

The system of equations (4) - (6) was solved numerically using the finite difference method with parameters corresponding to the studied experimental samples and given in Table 1.

Table S1. Parameters of the model (4) – (7)

| Parameter | Symbols | Values | Units |
| --- | --- | --- | --- |
| Thickness of the bottom dielectric film (TiO_2_) | $d_{B}$ | 30 | nm |
| Thickness of the top dielectric film (Al_2_O_3_) | $d_{T}$ | 5 | nm |
| Permittivity of the bottom dielectric film | $\varepsilon_{B}$ | 90 | – |
| Permittivity of the top dielectric film | $\varepsilon_{T}$ | 10 | – |
| Electron mobility in the active layer | $\mu_{n}$ | 5 | cm^2^/(V·s) |
| Effective mass of electrons in the active layer | $m_{n}$ | 0,6 | – |
| Difference between the bottom of the conduction band and energy level of the traps in the active layer | ${E_{C}-E}_{t}$ | 2 | eV |
| Hopping transport activation energy for $\mathcal{E}_{z}=0$ | $E_{a0}$ | 1,8 | eV |
| Hopping transport model parameter | $\varepsilon_{p}$ | 8⋅10^-3^ | eV |
| Spin degeneracy factor (statistical weight) of trap levels | $g$ | 2 | – |
| Resistivity of the bottom dielectric film | $\rho_{B}$ | 10^7^ | Ohm·cm |
| Temperature | *T* | 300 | K |

**Electrical properties at the nanoscale level in Pt-BE / TiO_2_ (30 nm) / Al_2_O_3_ (5 nm) / Pt-TE structures**

The results of optical measurements indicate that TiO_2_ film contains the anatase phase [Fig. S1] with the presence of amorphous phase. The relative phase content depends on the annealing temperature and the thickness of the film. The band gap of the TiO_2_ film corresponds to the anatase phase.


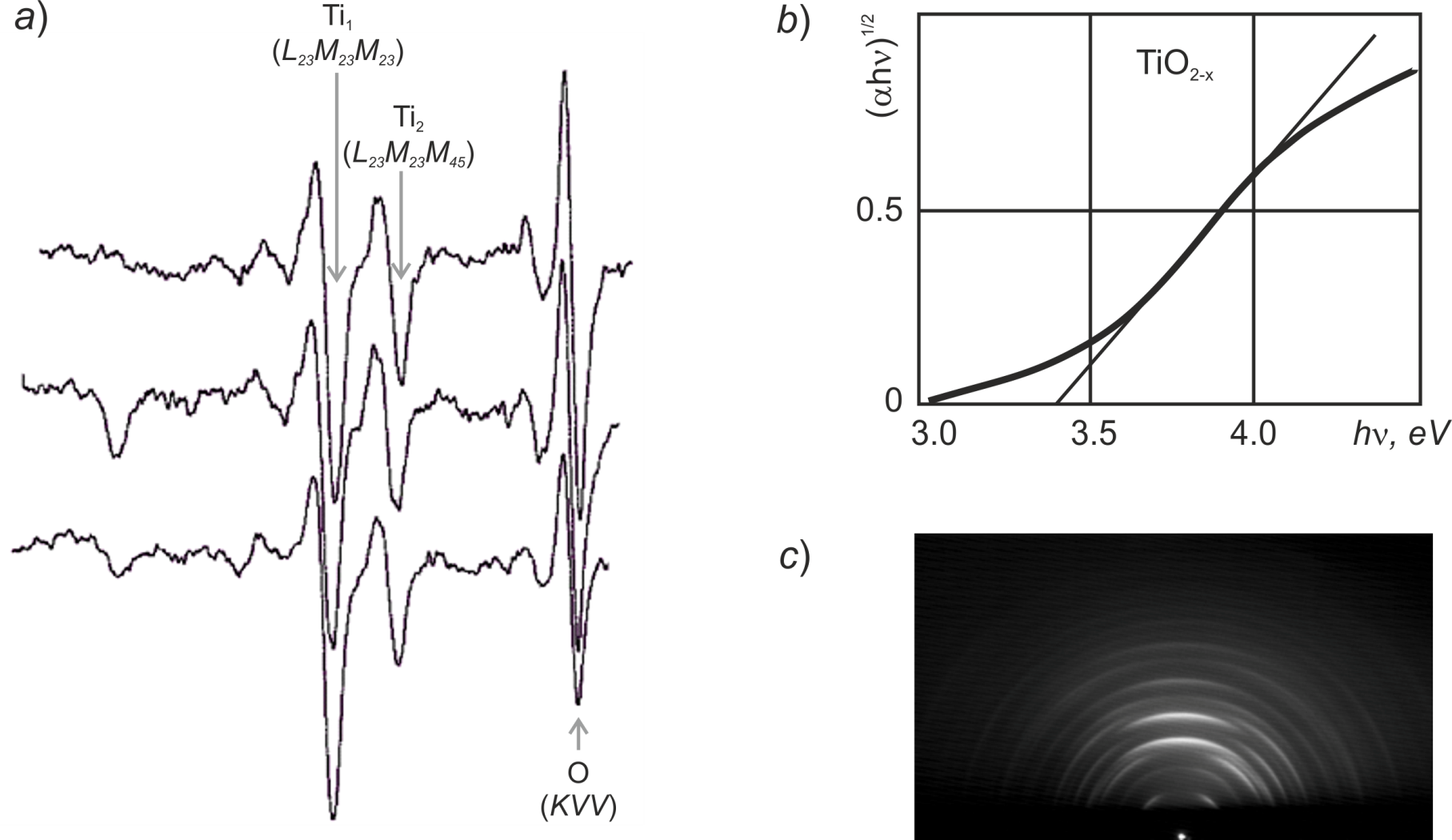


**Figure S1.** Optical absorption spectrum of TiO_2_ thin film grown on a sapphire substrate with Urbach-type edge

The results of topography measurements of TiO_2_/Al_2_O_3_ structure are shown in Fig. S2 and suggest that the reason for appearance of the grains on the topography of TiO_2_/Al_2_O_3_ bilayers is an inclusion of anatase phase in TiO_2_ layer [Andreeva, N. V., Chigirev, D. A., Kunitsyn, A. S., and Petrov, A. A. (2018). Reversible modification of electrical properties at the nanoscale level in bilayer oxide systems. IOP Conf. Ser.: Mater. Sci. Eng. 443, p. 012003. doi: 10.1088/1757-899X/443/1/012003].


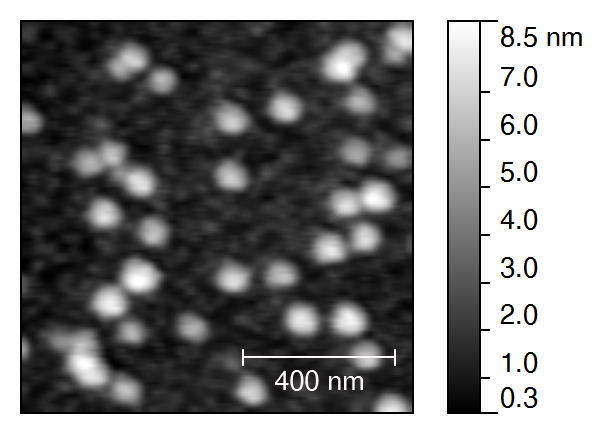


**Figure S2.** Topography of TiO_2_/Al_2_O_3_ (30 nm / 5 nm) bilayer surface

The results of measurements of the current distribution over the surface of TiO_2_ thin films [Fig. S3(a,b)] and TiO_2_/Al_2_O_3_ bilayers [Fig. S3(c,d)] suggest that areas of the samples associated with the grains are more conductive.

| *(a)* | *(b)* |
| --- | --- |
| 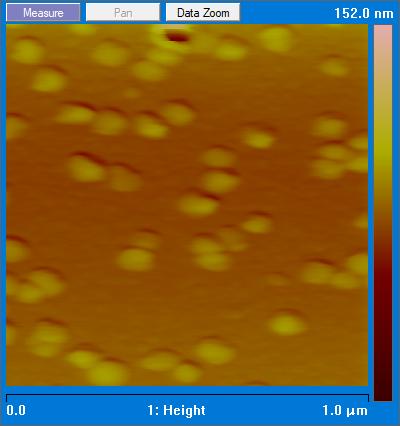 | 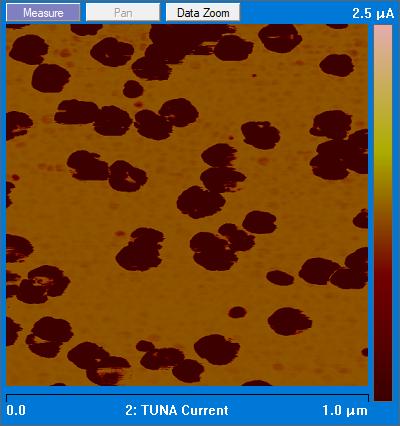 |
| *(c)* | *(d)* |
| 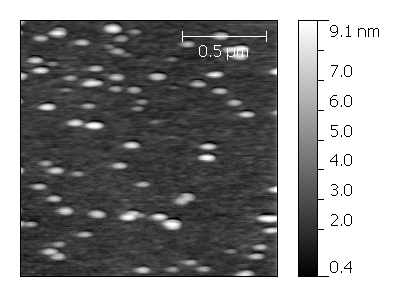 | 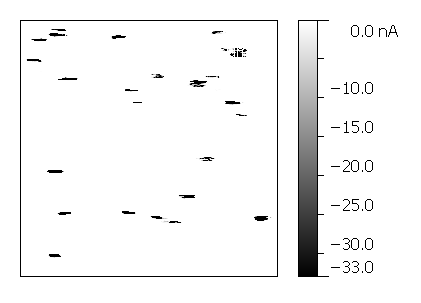 |

**Figure S3**. (a) the topography and (b) the current distribution over the surface of TiO_2_ (30 nm) obtained at bias voltage of -2 V applied between the conductive AFM tip and the bottom electrode of the structure; (c) the topography and (d) the current distribution over the surface of TiO_2_/Al_2_O_3_ (30 nm / 5 nm) obtained at bias voltage of -4 V applied between the conductive AFM tip and the bottom electrode of the structure.

Scanning with a bias applied between the AFM tip and the bottom electrode of these structures leads to reversible formation of conductive areas in the top aluminum oxide layer located directly above the anatase crystallites. These conductive areas could be switched back into the high resistance state by application of bias voltage of reverse polarity. We link an appearance of conductive areas in TiO_2_/Al_2_O_3_ bilayers with the possible reversible change of the conductivity of the Al_2_O_3_ layer, which we relate to multilevel resistive states in these structures.

**Influence of ALD temperature on the electrical properties of Al_2_O_3_ layer**

The influence of the deposition temperature on the electrical properties of ALD-Al_2_O_3_ thin layers was investigated in single-layer Pt-BE/ALD-Al_2_O_3_/Pt-TE structures. ALD of 10 nm-thick Al_2_O_3_ layer was carried out using trimethylaluminum and water vapor (H_2_O) at temperatures of 150°C - 290°C. Increasing the deposition temperature leads to an increase in the resistivity of the single-layer structure [Fig. S4]. The breakdown voltage for a given geometry of Pt-BE/ALD-Al_2_O_3_/Pt-TE structures deposited at 150°C is equal to 6-8 V; for structures deposited at 200°C it exceeds 20 V.


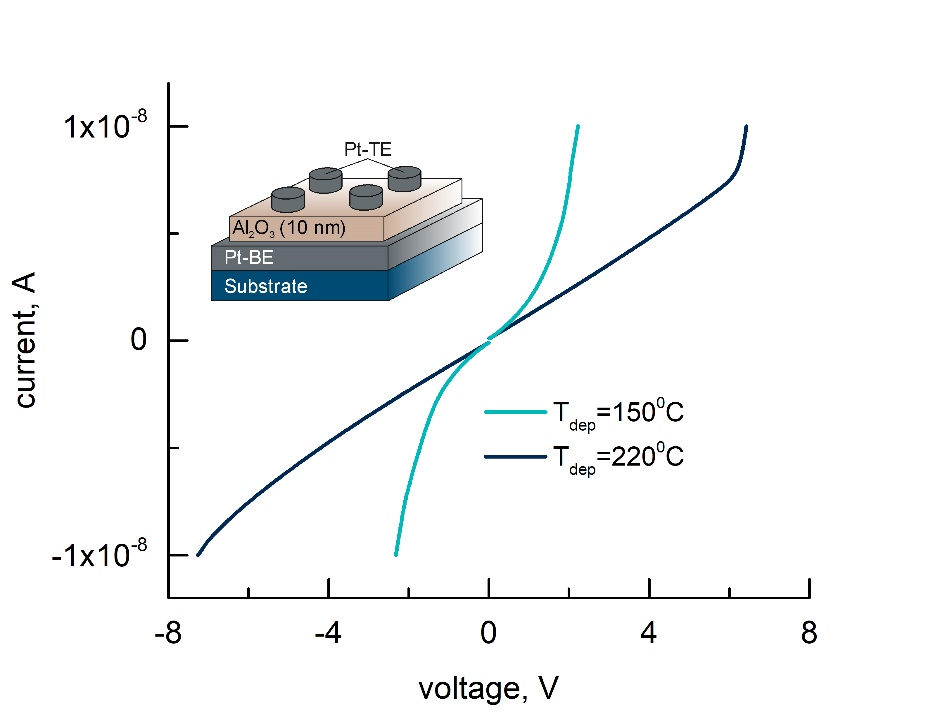


Figure S4. The experimental I-V characteristics of Pt/Al_2_O_3_/Pt structures with 10 nm –thick Al_2_O_3_ layer deposited at two different temperatures T_dep_ = 150°C and T_dep_ = 220°C

It is quite difficult to estimate the influence of the deposition temperature on the properties of Al_2_O_3_ in bilayer structures, because at ALD temperatures above 200°C, the phase composition of the titanium dioxide layer changes, which itself affects the electrophysical properties of the Pt/TiO_2_/Al_2_O_3_/Pt structures.

Nevertheless, we fabricated structures in which the bottom layer of titanium dioxide was deposited at 150°C and the top layer of aluminum oxide at a higher temperature. For structures with an aluminum oxide layer deposited at 150°C, the transition to the nonlinear part of I-V characteristics occurs at lower voltages, thus shifting the operating range of bilayer structures to a lower voltage range [Fig. S5(a)]. In addition, it should be noted that the transition to the nonlinear part manifests itself in the S-shaped I-V curve for the bilayer [Fig. S5(b)].

| *(a)* | *(b)* |
| --- | --- |
| 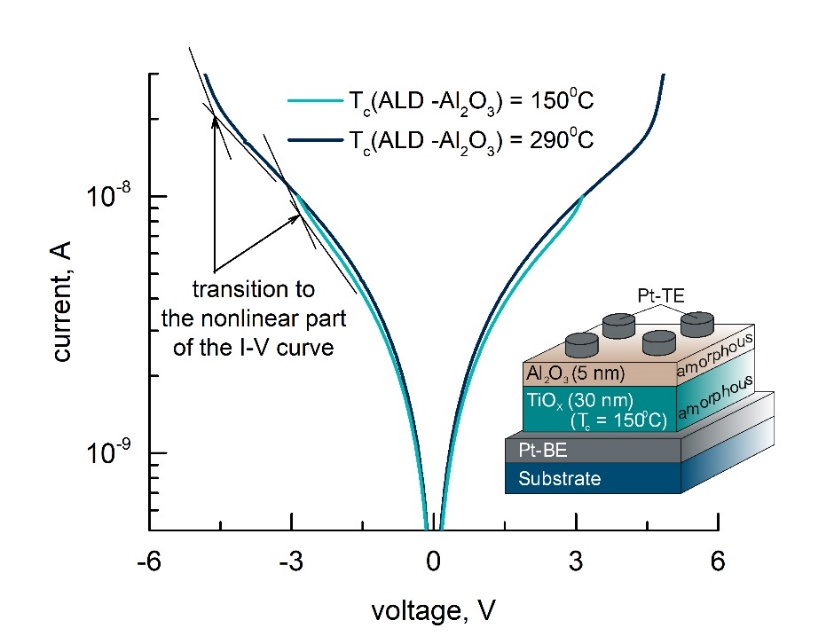 | 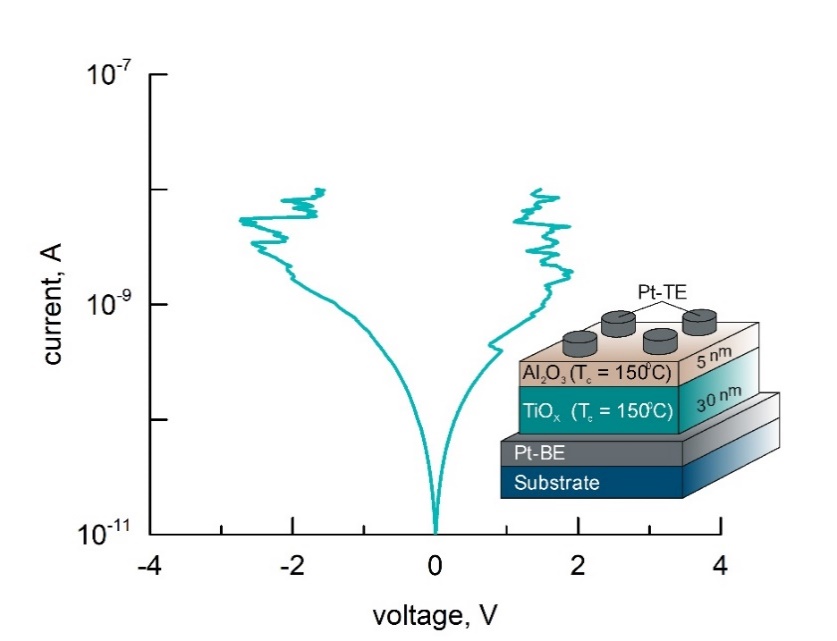 |

Figure S5. (a) The experimental I-V characteristics of Pt/ TiO_2_/Al_2_O_3_/Pt structures with ALD - Al_2_O_3_ layer deposited at two different temperatures T_dep_ = 150°C and T_dep_ = 290°C. (b) The experimental I-V characteristics of Pt/ TiO_2_/Al_2_O_3_/Pt structures with ALD - Al_2_O_3_ layer deposited at T_dep_ = 150°C measured in a current source mode
